# Anticancer, Antibacterial, Antioxidant, and DNA-Binding Study of Metal-Phenalenyl Complexes

**DOI:** 10.1155/2022/8453159

**Published:** 2022-04-14

**Authors:** Subhadeep Sen, Nilkanta Chowdhury, Tae-Wan Kim, Mohuya Paul, Dilip Debnath, Seob Jeon, Angshuman Bagchi, Jungkyun Im, Goutam Biswas

**Affiliations:** ^1^Department of Chemistry, Cooch Behar Panchanan Barma University, Panchanan Nagar, Vivekananda Street, Cooch Behar 736101, West Bengal, India; ^2^Department of Biochemistry and Biophysics, University of Kalyani, Nadia 741235, West Bengal, India; ^3^Department of Medical Life Science, Soonchunhyang University, Asan 31538, Republic of Korea; ^4^Department of Electronic Materials, Devices, and Equipment Engineering, Soonchunhyang University, Asan 31538, Republic of Korea; ^5^Department of Obstetrics and Gynecology, College of Medicine, Soonchunhyang University Cheonan Hospital, Cheonan 31151, Republic of Korea; ^6^Department of Chemical Engineering, Soonchunhyang University, Asan 31538, Republic of Korea

## Abstract

Phenalenyl (PLY)-based metal complexes are a new addition to the metal complex family. Various applications of metal-based phenalenyl complexes (metal-PLY) have been reported, such as catalyst, quantum spin simulators, spin electronic devices, and molecular conductors, but the biological significance of metal-PLY (metal = Co(II), Mn(III), Ni(II), Fe(III), and Al(III)) systems has yet to be explored. In this study, the anticancer properties of such complexes were investigated in ovarian cancer cells (SKOV3 and HEY A8), and the cytotoxicity was comparable to that of other platinum-based drugs. Antibacterial activity of the metal-PLY complexes against both gram-negative (*E. coli*) and gram-positive (*S. aureus*) bacteria was studied using a disk diffusion test and minimum inhibitory concentration (MIC) methods. All five metal-PLY complexes showed significant antibacterial activity against both bacterial strains. The antioxidant properties of metal-PLY complexes were evaluated following the 2,2-diphenyl-1-picrylhydrazyl (DPPH) scavenging method and were acceptable. The DNA-binding properties of these metal-PLY complexes were investigated using absorption spectroscopy, fluorescence spectroscopy, viscosity measurements, and thermal denaturation methods. Experimental evidence revealed that the complexes bind to DNA through intercalation, and the molecular docking study supported this conclusion.

## 1. Introduction

Metal complexes are one of the oldest and most important topics in chemistry. From the first synthesis of Prussian blue in 1706 to the present, the importance of metal complex chemistry has persisted, and its applications have increased tremendously [1, 2]. Metal complexes have a wide range of applications in medicine [3, 4], catalysts [5], coordination polymers [6], indicators [7], antibiotics [8], antioxidants [9], and anti-inflammatory agents [10]. They also have biological importance as they are also present as heme groups in hemoglobin, myoglobin, and cytochromes [11, 12] and as chlorophyll in the chloroplast [13]. Hence, metal complexes have emerged as a pivotal topic for research. A large number of organic compounds form metal chelates (e.g., Ni(DMG)_2_ and Cu(acac)_2_) [14, 15]. PLY is one of the chelate ligands containing polycyclic aromatic hydrocarbons having three six-membered conjugated fused rings with an odd number of carbon atoms [16]. The understanding of the chemistry of PLY is relatively new, and it is an emerging area for synthetic researchers. The derivatives of PLY can bind easily to form stable chelate complexes with metals such as Al(III) [17], Mn(III) [18], Co(II) [19], Fe(III) [20], Pt(II) [21], and Ni(II) [22] and metalloids such as boron [23]. These derivatives form stable complexes, which are extensively used as catalysts [18, 24], anticancer drugs [21], quantum spin simulators [25], spin electronic devices [26], and molecular conductors [27].

At present, the world is in the midst of a pandemic. Although most people are focused on fighting ailments, we must not forget about our old foe, cancer. In 2018, there were 18.1 million new cancer diagnoses and 9.6 million cancer deaths, according to a report released on September 12 by the International Agency for Research on Cancer (IARC) [28]. Therefore, interest in eliminating this catastrophic disease is increasing. Since ancient times, metal compounds have been successfully used to treat a variety of diseases. Many chemical species have been produced and investigated as anticancer medications in the modern age, but the era of metal-based anticancer drugs began with the discovery of the platinum (II) complex cisplatin. Over time, both cisplatin and other platinum-based drugs such as carboplatin and oxaliplatin have become widely used for cancer treatment. The enormous success of cisplatin and its derivatives as anticancer agents has sparked the development of new types of metal-based compounds to combat cancer. Despite some drawbacks such as solubility issues, nontargeted action, and inactivation in biological environments by reducing agents such as metallothioneins [29], the use of such metal complexes has been successful in anticancer drugs. A seminal work was written on Pt-PLY complexes, which exhibited remarkable anticancer properties [21]. Thus, we speculate that other metal-phenalenyl complexes will have anticancer properties and need to be investigated both as anticancer agents and in terms of their biological activities, such as antibacterial, antioxidant, and DNA-binding activity.

The role of antibiotics is to provide protection against bacterial infections and to keep humans healthier and free from diseases. Extensive use of antibiotics has led the microorganisms to develop resistance against antibiotics [30]. With the evolution of new resistant bacterial strains, the mortality rates associated with bacterial infections are increasing. Metal complexes have played a vital role in the field of antibiotics. There is a wide range of such compounds that have decent antibacterial activity [31, 32].

During normal metabolic processes, reactive oxygen species (ROS) are generated in the human body [33, 34]. These free radicals can oxidize biomolecules, damage cells, and initiate degenerative diseases [35]. There are some enzymes (such as superoxide dismutase and catalase [36]) and organic molecules (such as flavonoids [37] and vitamin C [38]) that are well known as antioxidants for their protection against free radical-mediated damage within organisms. In addition to these naturally occurring small molecules, new therapeutic agents need to be developed to inhibit free radical damage. There is a variety of reported metal complexes that show decent antioxidant properties by scavenging free radicals [9, 39].

Since the discovery of cisplatin, DNA-metal complex interactions have gained a lot of attention and become a promising field of research due to the possible applications of metal complexes as anticancer or antitumor agents [28]. There are a large number of metal complexes reported to interact with DNA by intercalation or through weak interactions such as *π*-*π* stacking and ionic interactions [40, 41]. Thus, the synthesis and screening of complexes with DNA-binding ability, anticancer activity, antioxidant properties, and antibacterial properties would be of substantial biological importance. Previously, complexes of Co(II), Mn(III), Ni(II), Fe(III), and Al(III) were reported to show anticancer, antioxidant, and antibacterial activity [39, 42–46]. Therefore, we speculate that complexes of Co(II), Mn(III), Ni(II), Fe(III), and Al(III) with PLY derivatives will show anticancer, antibacterial, and antioxidant activity and can interact with DNA. Although various such activities of the metal-PLY complexes have been demonstrated, these in particular have yet to be reported. These findings prompted us to start a project on metal-PLY complexes, involving an investigation of their DNA-binding, anticancer, antibacterial, and antioxidant properties.

## 2. Experimental Studies

### 2.1. Materials

Cobalt acetate (Co(OAc)_2_ 4H_2_O), 9-hydroxyphenalenone, manganese chloride (MnCl_2_∙4H_2_O), aluminum chloride (AlCl_3_), nickel acetate (Ni(OAc)_2_ 4H_2_O), anhydrous FeCl_3_, ascorbic acid, 2,2-diphenyl-1-picrylhydrazyl (DPPH), and Tris buffer were purchased from Sigma-Aldrich, India. Salmon sperm DNA (SS-DNA) was purchased from SRL, India. All solvents including dimethylformamide (DMF), ethanol (EtOH), methanol (MeOH), dichloromethane (DCM), acetonitrile (CH_3_CN), dimethyl sulfoxide (DMSO), and THF were purchased from a local supplier and dried when necessary. All the UV-Vis measurements were performed using a Thermo Scientific Evolution 201 UV-Vis spectrophotometer with a 1 cm path length quartz cuvette (purchased from TUV Company).

### 2.2. Synthesis of Metal-PLY Complexes **(1**–**5)**

**Figure 1:**
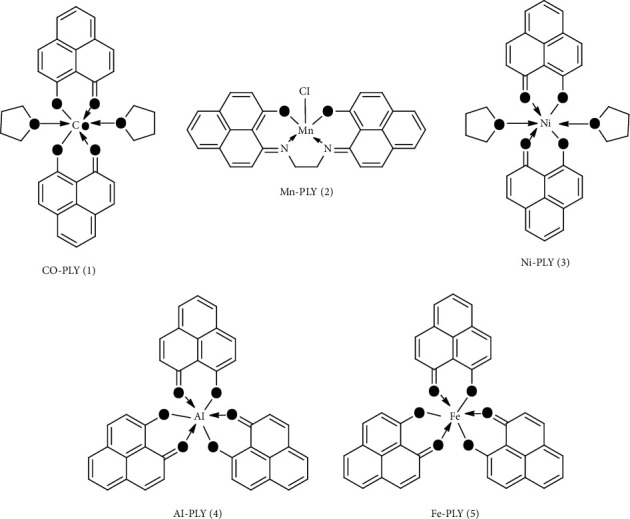
Structures of the metal-PLY complexes.

All metal-PLY complexes **(1**–**5)** (Figure 1) were prepared using synthetic protocols similar to those reported in the literature [17–20, 22]. The synthesized complexes were recrystallized and characterized by UV-visible spectrometry and thin-layer chromatography. Complexes showed a single band in thin-layer chromatography. The ligands were characterized using ^1^H-NMR spectroscopy (S2). The formation of the complexes was further confirmed by matching the UV-visible and mass spectra with those in the reported literature, the purity was confirmed by elementary analysis (S3–S9), and the hydrolytic behavior of the Mn-PLY **2** complex was evaluated using UV-Vis spectroscopy (S10) (provided in the supporting information).

### 2.3. Anticancer Effect by MTT Assay

SKOV3 and HEY A8 cells were seeded into 96-well plates at a concentration of 4.5 × 10^3^ cells/well in RPMI 1640. After 24 h, the cell medium was changed to non-serum RPMI 1640. Within 24 h, the cells were treated with metal-PLY complexes at different concentration ranges from 0 to 64 *μ*M. The metal-PLY complexes were dissolved in non-serum media containing 3.75% (v/v) DMSO for complete solubilization. The treated cells were incubated for 24 h and washed with cold PBS three times. The MTT reagent (3-(4,5-dimethylthiazol-2-yl)-2,5-diphenyltetrazolium bromide, 0.5 mg/ml) was added to cells and incubated for 4 h. The resulting formazan dye was quantified by measuring the absorbance of the samples at 540 nm to determine cell viability. As positive controls, paclitaxel, a tetracyclic diterpenoid (trade name Taxol), and carboplatin, which are well-known anticancer drugs, were dissolved in the media with the same DMSO content. Carboplatin is a platinum-based anticancer drug that is primarily used to treat ovarian cancer and was developed to overcome resistance to cisplatin in cells. Then, the solutions were added to the cells following the above protocol. Each experiment was performed in triplicate.

### 2.4. Antibacterial Study

#### 2.4.1. Bacteria Culture Preparation

A gram-negative bacterial strain of *E. coli* (ATCC25922) and a gram-positive bacterial strain of *S. aureus* (ATCC29213) were grown in Luria Broth (LB) medium at 37°C for 24 h, and the optical density (OD) was normalized to that of a 0.5 McFarland standard.

#### 2.4.2. Disk Diffusion Test

100 *μ*L of each bacterial cell suspension (0.5 McFarland standard) was spread over the LB agar plate and allowed to dry for 10 minutes. The plate was covered with four sterile paper disks (5 mm), onto which 10 *μ*L of each metal-PLY complex **(1**–**5)** solution in DMSO (2.0 mg/mL, 1.0 mg/mL, and 0.5 mg/mL) was poured. As a negative control, 10 *μ*L DMSO solution was added to the fourth disk. Chloramphenicol was used as a positive control at the same concentrations. After being sealed with parafilm and incubated at 37°C for 24 h, the inhibition zones in each plate were measured [47–50].

#### 2.4.3. Minimum Inhibitory Concentration (MIC)

The MIC of metal-PLY complexes **(1**–**5)** was measured using microliter wells and a broth dilution technique. LB (50 *μ*L) was dispensed in each well of columns 1–10. Then, 100 *μ*L of standardized bacteria inoculum was placed in column 11 (positive control), and 100 *μ*L LB was placed in column 12 for monitoring the sterility of the experiment (negative control). The positive control sample included 100 *μ*l of bacteria inoculum. Then, 50 *μ*l of a serially diluted metal-PLY complex solution in DMSO was added to columns 1–10 with concentrations ranging from 8 mg/mL to 0.015 mg/mL. Subsequently, a standardized bacterial culture (1 × 10^5^ cfu/mL) was added to each well from columns 1–10 and incubated for 24 h at 37°C. The MIC was determined at the lowest concentration of no visible growth of bacteria [47].

### 2.5. Antioxidant Assay

The most commonly used technique to evaluate antioxidant activity is the scavenging assay with DPPH (stable free radical), which has a significant absorption band at 517 nm and can receive electrons or hydrogen atoms from the antioxidant to be reduced. After reduction, the absorbance at 517 nm changed, allowing the scavenging activity to be evaluated [51]. The antioxidant activity of metal complexes **(1**–**5)** was examined by the reaction with DPPH. Each metal-PLY complex was tested at different concentrations (0.16 mg/mL, 0.08 mg/mL, 0.04 mg/mL, and 0.02 mg/mL) in 9 mL DMSO, and 1 mL of 0.1 mM DPPH solution was added to each solution. For the control experiment, only 9 mL DMSO and 1 mL DPPH solution were mixed. Metal-PLY complex solutions in DMSO were measured at the same concentration as blanks. For incubation, these combinations were held at room temperature for 30 minutes in the dark. The scavenging efficacy of each solution was measured after incubation as the absorbance of each solution was at 517 nm. Vitamin C (ascorbic acid) was employed as a standard antioxidant (positive control) [48, 52], and the experiment was repeated three times.

Percentages of DPPH scavenging ability of the metal-PLY complexes were determined according to the following equation:

(1) $$\begin{array}{c} \text{Scavenging activity}\left(\text{\%}\right)=\frac{A_{0}-A_{s}}{A_{0}}\times 100, \end{array}$$

where *A*_0_ is the absorbance of the control (DPPH + DMSO) and *A*_*s*_ is the absorbance of the sample solutions (sample in DMSO + DPPH solution) excluding that of the blank.

The IC_50_ value (at which concentration the complex showed 50% scavenging activity) was calculated from the graph.

### 2.6. DNA-Binding Study

SS-DNA-binding experiments with metal complexes **(1**–**5)** were conducted in 10 mM Tris-HCl/50 mM NaCl (pH = 7.5) buffer solution using universally employed electronic absorption spectroscopy. The quality of SS-DNA was tested before the binding experiment by measuring the absorbance ratio at 260 and 280 nm in Tris-HCl-NaCl buffer solution. This ratio was 1.81, suggesting that the DNA was suitably pure. The SS-DNA concentration was calculated using the absorbance at 260 nm and a molar extinction value (*ℰ*) of 6600 M^−1^cm^−1^ [53]. The absorption titration experiment was carried out using 10 *μ*L aliquots of DNA solution added sequentially to a fixed amount of metal complex. For each sample, the mixed solutions were allowed to incubate for 2 minutes before the absorption spectra were recorded. The binding constant (*K*_*b*_) of the SS-DNA-PLY complexes was calculated using the following equation [54]:

(2) $$\begin{array}{c} \frac{\left[\text{DNA}\right]}{E_{a}-E_{f}}=\frac{\left[\text{DNA}\right]}{E_{b}-E_{f}}+\frac{1}{K_{b}\left(E_{b}-E_{f}\right)}, \end{array}$$

where (DNA) is the concentration of DNA in the base pairs; *ℰ*_*a*_, *ℰ*_*f*_, and *ℰ*_*b*_ are the extinction coefficients of the free metal complex, each sample of DNA and metal complex, and the metal complex in its completely bound state, respectively.

### 2.7. Viscosity Measurements

The spectroscopic data provided evidence for the interaction of metal-PLY complexes with SS-DNA. To support the spectroscopic data, we measured the viscosity. Changing the length of the DNA should increase viscosity, and the viscosity measurement provides good evidence for the interaction. A hydrodynamic measurement, such as viscosity, is a significant tool in evaluating the binding mode between tested drugs and DNA because it adds high accuracy to any change in DNA length. Viscosity experiments were conducted using an Ostwald viscometer at 25.0 ± 0.1°C. Titrations were performed for the compounds, and each compound was added to the DNA solution. Data were presented as (*η*/*η*_0_)^1/3^ versus the ratio of the concentration of the compound to DNA, where *η* is the viscosity of DNA in the presence of the compound and *η*_0_ is the viscosity of free DNA [40, 55].

### 2.8. Thermal Denaturation

The thermal stability of DNA-metal-PLY complex was measured as a function of temperature. The change in absorbance at 260 nm with temperature was monitored from 30°C to 90°C at a 1°C per minute scan rate for a fixed ratio of (metal-PLY complex)/(DNA). Accordingly, the DNA melting temperature (*T*_m_) was recorded [41, 56].

### 2.9. Fluorescence Quenching Study

Ethidium bromide (EB) intercalates between adjacent base pairs of DNA and exhibits a strong fluorescence band. EB fluorescence can be quenched by adding a second molecule that binds to the DNA. The extent of EB fluorescence quenching can be used to indirectly calculate the binding constant of the metal-PLY complex to SS-DNA. Competitive binding assays were conducted by adding aliquots of metal-PLY complexes to a fixed (DNA)/(EB) ratio of 10. The fluorescence spectra of EB were measured using an excitation wavelength of 525 nm, and the emission spectra were recorded between 550 and 750 nm. The spectra were analyzed according to the classical Stern–Volmer equation:

(3) $$\begin{array}{c} \frac{I_{0}}{I}=1+K_{\text{sv}}\left[Q\right], \end{array}$$

where *I*_0_ and *I* are the fluorescence intensities at 595 nm in the absence and presence of the quencher, respectively; *K*_sv_ is the linear Stern–Volmer quenching constant; and (Q) is the ratio of concentrations of quencher and SS-DNA [41, 57].

### 2.10. Molecular Docking Studies

Molecular docking simulations were performed to study the binding interactions between the ligands and the DNA. Initially, the coordinates of the DNA were extracted from the Protein Data Bank using the PDB code 1BNA (sequence: d(CGCGAATTCGCG)2) [58]. To reduce steric clashes, energy minimizations of the DNA were performed using 100 cycles each of steepest descent, followed by conjugate gradient protocols in the Discovery Studio 2.5 (DS2.5) platform until the structure of the DNA showed an energy derivative of 0.001 kcal/mole. Then, the structures of the ligands in DS2.5 were generated and subjected to energy minimization to obtain the stable conformations of the ligands. The PatchDock server was used for docking studies with surface complementarity information to generate the docked structures [59]. To achieve a consensus result, the docking simulations were performed again using AutoDock Vina as well [60]. The two tools (PatchDock and AutoDock Vina) generated nearly identical docking poses. The docked complexes of the ligands and DNA were visualized in DS2.5. These complexes were again subjected to energy minimization following the same protocol as previous. Finally, the binding free energy values between the ligand and DNA in the complexes were calculated using DS2.5.

## 3. Results and Discussion

### 3.1. Anticancer Effects of Metal-PLY Complexes

**Figure 2:**
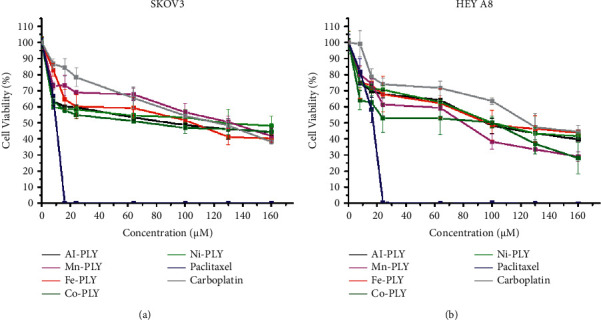
Cell viability measurement by MTT assay. (a) SKOV3 and (b) HEY A8 cell lines were treated with metal-PLY complexes for 24 h. Paclitaxel and carboplatin were added to cells as positive controls. All samples were dissolved in cell media containing 3.75% DMSO by volume for complete dissolution.

The cell viabilities after treatment of metal-PLY complexes and anticancer medicines were examined by an MTT assay to assess the cytotoxicity of metal-PLY complexes in comparison with those of paclitaxel and carboplatin. All the compounds were dissolved in cell media containing 3.75% (v/v) DMSO to dissolve samples completely. A DMSO content in cell media less than 5% is generally not harmful to the cells. The MTT assay showed that the metal-PLY complexes began to exhibit moderate cytotoxicity in both cell lines at a concentration of more than 20 *μ*M (Figure 2). All the metal-PLY complexes decreased cell viability in a similar pattern, implying they have comparable cytotoxicity toward SW480 and HEY A8 cells. When the metal complexes were treated at 160 *μ*M, the average cell viability of SW480 was 43% and that of HEY A8 was 37%. Paclitaxel and carboplatin were used as positive controls as approved anticancer drugs. As expected, paclitaxel showed the greatest cytotoxicity, demonstrating complete death of SKOV3 cells at 16 *μ*M of paclitaxel and of HEY A8 cells at 24 *μ*M. Carboplatin, which is an antineoplastic drug having platinum in the center with oxalate and diamino ligands and can be regarded as a metal complex, showed similar cytotoxicity to metal-PLY complexes. According to the cell viability values, the IC_50_ values of the samples are listed in Table 1. These results confirm that the metal-PLY complexes showed cytotoxicity toward ovarian cancer cells similar to that of carboplatin.

### 3.2. Antibacterial Study

#### 3.2.1. Disk Diffusion Test

The antibacterial activity of the metal-PLY complexes was assessed in the disk diffusion test using the zone of inhibition (ZOI) value (Table 2).

All compounds **(1**–**5)** had significant antibacterial activity against gram-positive (*E*. *coli*) and gram-negative (*S*. *aureus*) bacterial strains (Table 2), although the efficacy of the metal-PLY complexes **(1**–**5)** against both depended on the concentration (Table 2). With the increasing concentration of the metal-PLY complexes, their antibacterial activity increased. Among all metal-PLY complexes, both Mn-PLY **2** and Ni-PLY **3** showed the maximum ZOI of 14 mm against *E*. *coli* at 2 mg/mL, whereas all three complexes (Co-PLY **1**, Mn-PLY **2,** and Ni-PLY **3**) showed the maximum ZOI of 12 mm at 2 mg/mL of *S*. *aureus* with respect to the negative control (DMSO). Tweedy's chelation theory, which states that antibacterial effects of complexes are related to chelation and the biological role of the metal ion, can explain the antibacterial activity of metal-PLY complexes. The chelates inhibit the microbial cells' ability to respire, preventing them from producing their own proteins and continuing to grow. As a result, the improvement in antibacterial activity as a function of chelation was validated [61]. During chelation, the metal ion's positive charge is partially shared by the ligand's donor groups, the metal ion's polarity is lowered, and the ability of complexes to cross cell membranes is improved [62].

#### 3.2.2. Minimum Inhibitory Concentration (MIC)

Table 3 shows the MIC values of metal-PLY complexes **(1**–**5)** against gram-positive and gram-negative bacteria.

**Figure 3:**
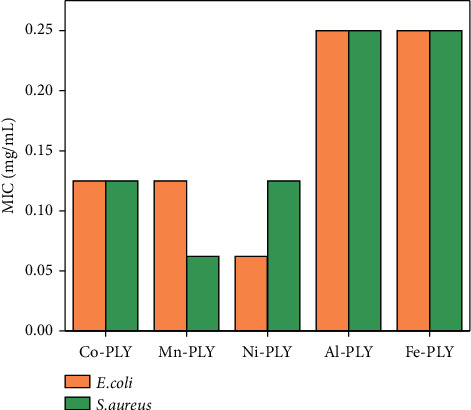
Graphical representation of the MIC values of the metal-PLY complexes **(1**–**5)** against *E*. *coli* and *S*. *aureus.*

Table 3 shows the similar MIC values of all metal-PLY complexes. For *E. coli,* the MIC values are in the order of Ni-PLY **3** > Mn-PLY **2**≈Co-PLY **1** > Al-PLY **4**≈Fe-PLY **5**. However, those against *S*. *aureus* are Mn-PLY **2** > Ni-PLY **3≈**Co-PLY **1** > Al-PLY **4≈**Fe-PLY **5** as shown in Figure 3. The negative control did not show any bacterial growth.

The MIC values of different reported metal complexes are tabulated (Table 4), and MIC values of the metal-PLY complexes were comparable with those of other reported metal complexes.

Table 4 shows that the Ni-PLY **3** and Co-PLY **1** complexes had better efficacy than those of other reported Ni and Co complexes, whereas Fe-PLY **5** and Mn-PLY **2** showed a higher MIC (lower efficacy) than those of other iron and manganese complexes against both bacterial strains.

### 3.3. Antioxidant Assay

#### 3.3.1. DPPH Scavenging Activity

**Figure 4:**
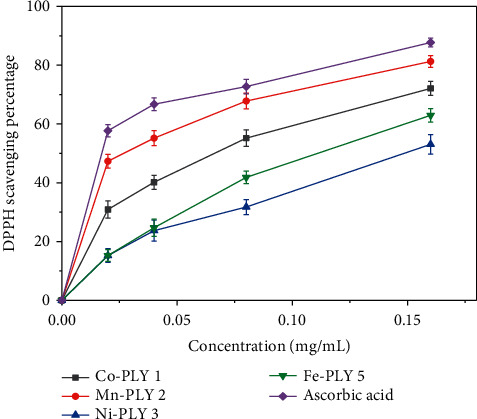
Graphical representation of the DPPH scavenging activity of metal-PLY complexes.

The antioxidant property of the metal-PLY complexes **(1**–**5)** and ascorbic acid (standard) was studied in terms of DPPH scavenging ability.  Figure 4 shows that the free radical scavenging depended upon concentration. The scavenging percentage also increased with concentration. The Mn-PLY **2** complex, which was closest to standard ascorbic acid among the metal-PLY complexes, had the best antioxidant properties. For the metal-PLY complexes (Table 5), we concluded that the IC_50_ values were in the order of ascorbic acid (standard) > Mn-PLY **2** > Co-PLY **1** > Fe-PLY **5** > Ni-PLY **3**. Mn-PLY **2** exhibited better anti-oxidant properties than other metal-PLY complexes, which could be due to the Mn^3+^/Mn^2+^ redox potential. Alternatively, ligands of the Mn-PLY **2** complex might have enhanced the electron donation ability of the complex. The Al (III) complex (Al-PLY**4**) did not show any DPPH scavenging activity, which might be due to the lack of variable oxidation states of aluminum.

### 3.4. DNA-Binding Study

**Figure 5:**
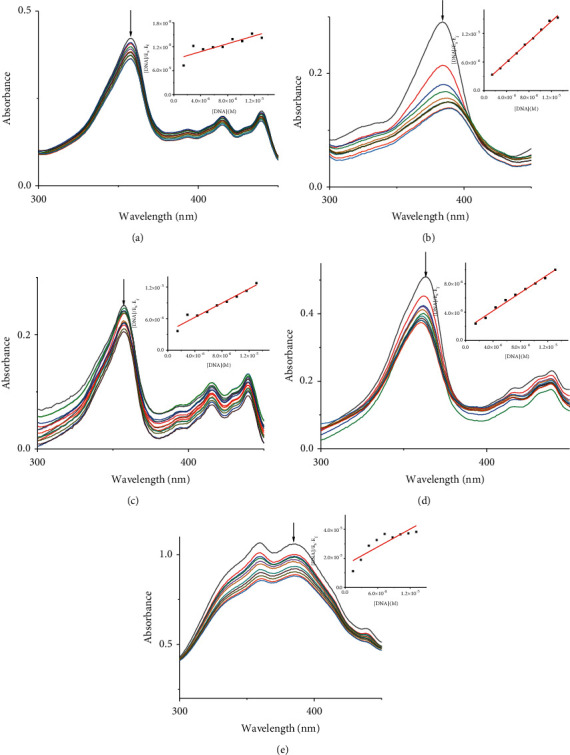
Absorption spectra of (a) Co-PLY **1**, (b) Mn-PLY **2**, (c) Ni-PLY **3,** (d) Al-PLY **4**, and (e) Fe-PLY **5** complexes with the increasing amounts of SS-DNA (0–100 *μ*L). The arrow shows that the absorption changes as the amount of DNA increases. Inset: (DNA)/(*ℰ*_*a*_−*ℰ*_*f*_) versus (DNA) curve.

Absorption spectroscopy is one of the most frequently used techniques for evaluating the binding of metal complexes to DNA. Because of the strong *π*-*π* stacking interactions or dipolar interactions between the aromatic chromophore of the ligand and DNA base pairs, the interaction between the metal complex and DNA is accompanied by an alteration in absorbance (hypochromism or hyperchromism) and a shift in *λ*_max_ (blueshift or redshift). Absorption spectra of metal-PLY complexes in the absence and presence of SS-DNA are provided in Figure 5. As the amount of SS-DNA increased, the absorption of complexes **(1**–**5)** exhibited hypochromism with blueshifts or redshifts. Co-PLY **1** and Al-PLY **4** complexes showed blueshifts, whereas Mn-PLY **2**, Ni-PLY **3**, and Fe-PLY **5** exhibited redshifts. The extent of the redshift or blueshift in *λ*_max_ is correlated with the DNA-binding strength. The Mn-PLY **2** complex showed a maximum 4 nm redshift in absorption spectra upon binding to SS-DNA, which is reflected in the corresponding Mn-PLY-DNA-binding constant (*K*_b_) value (5.37 × 10^5^). The binding ability of the metal complexes follows the order Mn-PLY **2** > Co-PLY **1** > Al-PLY **4** > Ni-PLY **3** > Fe-PLY **5** as shown in Table 6.

### 3.5. Viscosity Experiments

**Figure 6:**
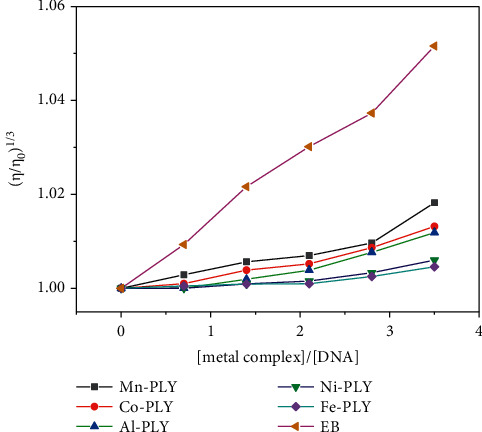
Effect of increasing amounts of the metal-PLY complexes on the relative viscosity of SS-DNA.

The viscosity of DNA solutions was measured using various dilutions of the compounds (compounds **1–5**) at a constant concentration of DNA solution.  Figure 6 depicts the effect of increasing compound concentrations on DNA viscosity. When a complex attaches to DNA in the traditional intercalative mode, the DNA base pairs are separated to accommodate the binding complex, resulting in lengthening of the DNA helix and increased DNA viscosity. The viscosity of DNA solution is reduced when a compound attaches in a nonclassical intercalative way. The relative viscosity of complexes increases continuously as the concentration of complexes increases, comparable to the behavior of ethidium bromide [40, 65, 66]. The increased degree of viscosity follows the order EB > Mn-PLY **2** > Co-PLY **1** > Al-PLY **4** > Ni-PLY **3** > Fe-PLY **5**. The increase in viscosity indicates that the compounds bind to DNA by intercalation, which is supported by UV-Vis spectral data.

### 3.6. Thermal Denaturation

Thermal denaturation studies were carried out to support the absorption titration data. The hydrogen bond energy that held two helices of DNA together decreased with increasing temperature. As a result, thermal energy causes structural alterations in DNA. Due to the breakage of hydrogen bonds at higher temperatures, double-stranded DNA denatured to a single-stranded structure. The melting temperature “*T*_m_” is the temperature at which 50% of molecules are denatured. Intercalation of compounds stabilizes the double-helix structure of DNA by stacking interactions, and the *T*_m_ value increases [67, 68]. The melting temperature (*T*_m_) of SS-DNA was 76.4°C but increased to 78.29, 78.37, 77.59, 78.11, and 77.27°C in the presence of Co-PLY **1**, Mn-PLY **2,** Ni-PLY **3**, Al-PLY **4,** and Fe-PLY **5,** respectively. These *T*_m_ values support the binding constants obtained from adsorption titration. According to the findings, chemicals interact efficiently with DNA via an intercalation method.

### 3.7. Fluorescence Quenching Study

**Figure 7:**
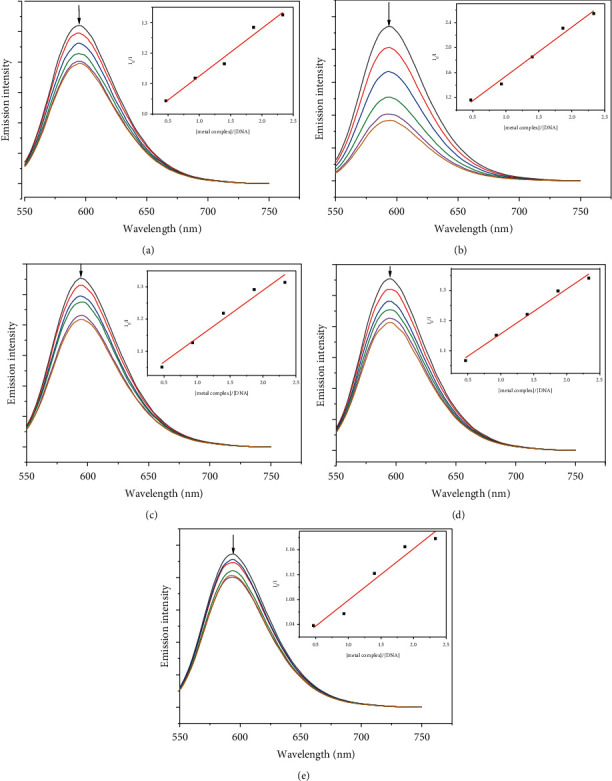
Emission spectra of EB bound to DNA in the presence of the complexes (a) Co-PLY **1**, (b) Mn-PLY **2**, (c) Ni-PLY **3,** (d) Al-PLY **4**, and (e) Fe-PLY **5**. The arrow shows the intensity changes upon increasing concentrations of the complex (0, 0.23, 0.46, 0.70, 0.93, and 1.16 mM, respectively). Inset: (plots of I_0_/I versus (metal complex)/(DNA)).

Due to the high intercalation of EB between DNA base pairs, EB exhibited strong fluorescence in the presence of DNA, and EB fluorescence can be quenched by adding a second EB molecule [57]. The extent of binding of the second molecule to DNA can be indirectly determined by the quenching of EB fluorescence.  Figure 7 shows the emission spectra of EB bound to DNA in the absence and presence of complexes. When complexes **1**–**5** were added to EB-DNA, the emission intensity decreased significantly, showing that the complexes compete with EB for DNA binding [41, 69].  Figure 7 shows the fluorescence quenching curves of EB bound to DNA by the metal-PLY complexes. The quenching constants were estimated from the linear Stern–Volmer plots and suggest that all five complexes bind to DNA. The K_sv_ values in the linear fit plots of I_0_/I vs. (metal complex)/(DNA) were 0.1561, 0.7824, 0.1481, 0.1491, and 0.0829 for Co-PLY **1**, Mn-PLY **2,** Ni-PLY **3**, Al-PLY **4,** and Fe-PLY **5,** respectively. Based on the *K*_sv_ values, the binding strength order varies in the order of Mn-PLY **2** > Co-PLY **1** > Al-PLY **4** > Ni-PLY **3** > Fe-PLY **5**, which is in good agreement with the UV-Vis binding data.

### 3.8. Molecular Docking Study

**Figure 8:**
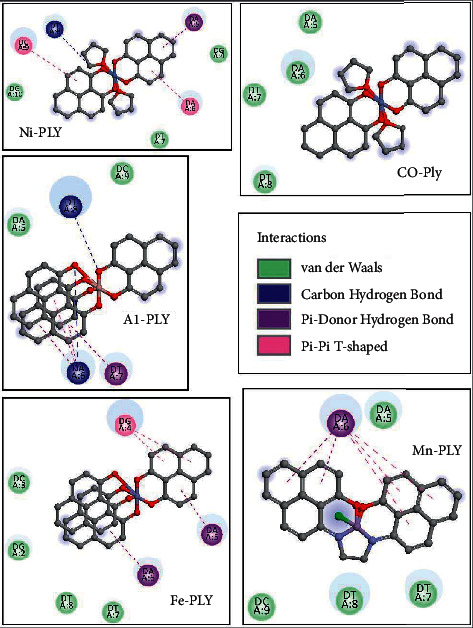
Binding interactions of metal-PLY with SS-DNA.

Docking studies revealed that the interaction of metal-PLY complexes with SS-DNA is thermodynamically favorable. We calculated the binding free energy values of the complexes and observed that Mn-PLY-SS-DNA complex has the best binding interactions among all the ligands as observed from the spectrophotometric studies. The binding free energy values of the other complexes are presented in Table 7. However, the energy differences between the complexes as obtained from the docking studies are not very significant. Therefore, these complexes seem to follow the results of the spectroscopic analysis. However, there are some changes in the interaction energy values of the complexes. The apparent anomaly between these values as obtained from spectroscopic data and docking studies might be due to the entropic factors, which might play a predominant role in the metal-PLY-DNA interactions. The docking study primarily emphasizes the interaction energy between the metal-PLY complexes **(1**–**5)** and DNA (Figure 8). With the help of docking studies, it could be concluded that the metal complexes have the abilities to bind the DNA. This would further testify to the binding efficiencies of ligands with SS-DNA. Furthermore, an overview of the thermodynamic parameters of binding, such as the binding free energy values of interactions between ligands and DNA, could be retrieved from the analyses of the docked complexes. UV-visible spectroscopy mainly provides information regarding the microenvironment of the chromophore. During intercalation of metal-PLY complexes with DNA, very often changes in the microenvironment of the chromophore occur by releasing the DNA-bound water molecules and ions and also by conformational changes in the chromophore. Thus, a change in the chromophore microenvironment is indirectly related to the entropic factor. The strong interaction of metal-PLY complexes may release DNA-bound water molecules or ions, which cause an increase in entropy. The increase in entropy after binding the PLY complexes to DNA could result in decreasing the binding free energy values according to the Gibbs free energy equation, i.e., ΔG = ΔH − TΔS (ΔG = change in free energy, ΔH = change in enthalpy, *T* = temperature, ΔS = change in entropy) and resulting in higher binding constant value.

## 4. Conclusion

In summary, anticancer, antibacterial, antioxidant, and DNA-binding activities of previously reported metal-PLY complexes **(1**–**5)** were examined. All synthesized metal-PLY complexes Co-PLY **1**, Mn-PLY **2**, Ni-PLY **3**, Al-PLY **4,** and Fe-PLY **5** showed moderate cytotoxicity against ovarian cancer cells (SKOV3 and HEY A8) even in the presence of DMSO in cell media. The chemical and biological findings of this study demonstrated that these complexes can show activity in human cancer cells in vitro in a reasonable range of concentrations, indicating these complexes as promising candidates as anticancer agents. Further studies are needed to assess pharmacological properties in vivo and to elucidate the actual mechanism of biological activity. The metal-PLY complexes also satisfied the demand of being an excellent antibacterial agent by exhibiting significant results against tested bacterial strains. Except for the Al-PLY **4** complex, the others showed significant DPPH scavenging activity, and the experimental data revealed that they could be good antioxidants. Therefore, the metal-PLY complexes can be a suitable substitution for antibiotics and an antioxidant agent. DNA-binding studies of the complexes were investigated by UV-Vis, fluorescence, thermal melting, and viscosity measurements. These studies confirm that the metal-PLY complexes **(1–5)** bind to SS-DNA with a decent binding constant, as determined by the absorption titration method. These results provide evidence that the metal-PLY complexes **(1–5)** have multifunctional properties and have potential practical applications, enhancing the potential benefit for further research on metal-PLY complexes **(1–5)** for evaluation of other biological properties such as antifungal and anti-inflammatory properties.

## Figures and Tables

**Table 1:** Cytotoxicity levels of metal-PLY complexes in two kinds of ovarian cancer cells.

Compound	IC_50_ (*μ*M)	
	SKOV3	HEY 48
Al-PLY	92.9	98.1
Fe-PLY	108.2	99.4
Ni-PLY	140.9	105.6
Mn-PLY	135.3	83.0
Co-PLY	77.6	104.4
Carboplatin	126.6	128.2
Paclitaxel	<20	<20

**Table 2:** ZOI values against *E. coli* and *S. aureus* of various metal-PLY complexes **(1**–**5)** and positive control chloramphenicol at different concentrations.

Metal-PLY complex	Concentration (mg/mL)	ZOI (mm)	
		*E*. *coli*	*S*. *aureus*
Co-PLY **1**	2.0	11	12
	1.0	9	11
	0.5	7	8
Mn-PLY **2**	2.0	14	12
	1.0	12	10
	0.5	8	8
Ni-PLY **3**	2.0	14	12
	1.0	13	10
	0.5	11	9
Al-PLY **4**	2.0	10	10
	1.0	9	8
	0.5	7	7
Fe-PLY **5**	2.0	12	10
	1.0	10	9
	0.5	8	7
Chloramphenicol	2.0	29	26
	1.0	27	22
	0.5	26	20

**Table 3:** MIC values of metal-PLY complexes **(1**–**5)** against *E*. *coli* and *S*. *aureus.*

Name of the metal-PLY complex	MIC value (mg/mL)	
	*E*. *coli*	*S*. *aureus*
Co-PLY **1**	0.125	0.125
Mn-PLY **2**	0.125	0.062
Ni-PLY **3**	0.062	0.125
Al-PLY **4**	0.250	0.250
Fe-PLY **5**	0.250	0.250

**Table 4:** MIC values of various metal complexes against *E*. *coli* and *S*. *aureus.*

Name of the complex	MIC (mg/mL)		Ref.
	*E*. *coli*	*S*. *aureus*	
Co(LL)	10.0	<10.0	[39]
CoL	0.250	0.250	[63]
Co-PLY **1**	0.125	0.125	Present study
C_21_H_24_NO_6_Mn	0.037	0.009	[46]
Mn-PLY **2**	0.125	0.062	Present study
NiL	0.500	0.250	[63]
Ni(LL)	<10	5	[39]
Ni-PLY **3**	0.062	0.125	Present study
Al-PLY **4**	0.250	0.250	Present study
[Fe_3_O(CH_3_COO)_6_(CH_3_COOH) (H_2_O)]Cl(MeImid)-(H_2_O)	0.075	0.065	[64]
[Fe(L_3_)(Cl)(H_2_O)]	0.058	0.056	[44]
Fe-PLY **5**	0.250	0.250	Present study

**Table 5:** DPPH scavenging capacity (IC_50_, mg/mL) of the standard (ascorbic acid) and the metal-PLY complexes.

Compound	Co-PLY 1	Mn-PLY 2	Ni-PLY 3	Fe-PLY 5	Ascorbic acid
IC_50_ (mg/mL)	0.086	0.059	0.143	0.115	0.042

**Table 6:** Experimental values of binding constant (*K*_b_) between SS-DNA and metal complex **(1**–**5)**.

Metal-PLY complex	Experimental binding constant **K**_*b*_ (M^−1^)
Co-PLY **1**	5.0 × 10^5^
Mn-PLY **2**	5.3 × 10^5^
Ni-PLY **3**	1.6 × 10^5^
Al-PLY **4**	3.1 × 10^5^
Fe-PLY **5**	1.0 × 10^5^

**Table 7:** Binding free energy values of metal-PLY complexes **(1**–**5)** with SS-DNA as calculated from molecular docking.

Metal-PLY complex	Theoretical binding energy (kcal/mol)
Co-PLY **1**	−26.427
Mn-PLY **2**	−50.444
Ni-PLY **3**	−45.514
Al-PLY **4**	−30.445
Fe-PLY **5**	−40.016

## Data Availability

The NMR data used to support the findings of this study have been deposited in the “Anticancer, Antibacterial, Antioxidant and DNA-Binding Study of Metal-Phenalenyl Complexes” repository doi: 10.5061/dryad.k98sf7m6p.

## Supplementary Materials

Supplementary Materials Page S1. ^1^H-NMR spectra of two ligands; pages S3–S9. Synthesis, UV-Vis, MS, and elemental analysis data of the metal-PLY **(1–5)** complexes; page S10. Hydrolytic behavior of Mn-PLY **(2)** complex. Available at the journal website.
