# Supplementary material for: Anticancer, Antibacterial, Antioxidant, and DNA-Binding Study of Metal-Phenalenyl Complexes
Source: Bioinorg Chem Appl. 2022 Apr 14;2022:8453159. doi: 10.1155/2022/8453159 (PMC9023202; doi:10.1155/2022/8453159)
Supplement: Supplementary Materials — Page S1. 1H-NMR spectra of two ligands; pages S3–S9. Synthesis, UV-Vis, MS, and elemental analysis data of the metal-PLY (1–5) complexes; page S10. Hydrolytic behavior of Mn-PLY (2) complex. Available at the journal website. [file 8453159.f1.docx]

Supporting Information

**Anticancer, Antibacterial, Antioxidant and DNA Binding Study of Metal-Phenalenyl Complexes**

Subhadeep Sen^1^, Nilkanta Chowdhury^2^, Tae-Wan Kim^3^, Mohuya Paul^4^, Dilip Debnath^1^, Seob Jeon^5^, Angshuman Bagchi^2^, Jungkyun Im^4,6^*****, Goutam Biswas^1^*****

^1^Department of Chemistry, Cooch Behar Panchanan Barma University, Panchanan Nagar, Vivekananda Street, Cooch Behar 736101, West Bengal

^2^Department of Biochemistry and Biophysics, University of Kalyani, Nadia 741235, West Bengal

^3^Department of Medical Life Science, Soonchunhyang University, Asan, 31538, Republic of Korea

^4^Department of Electronic Materials and Devices Engineering, Soonchunhyang University, Asan, 31538, Republic of Korea

^5^Department of Obstetrics and Gynecology, College of Medicine, Soonchunhyang University Cheonan Hospital, Cheonan, 31151, Republic of Korea

^6^Department of Chemical Engineering, Soonchunhyang University, Asan, 31538, Republic of Korea

UV-Vis spectroscopy was performed by Thermo Scientific Evolution 201 UV-vis spectrophotometer. Elemental analysis was studied in PerkinElmer 2400 series II CHNS/O analyzer. MS was studied by Waters Micromass QTof MicroTM.

**Content**

- **NMR spectra of two ligands …………………………….………S2**
- **Synthesis, UV-Vis, MS, and elemental analysis data of the metal PLY (1-5) complexes………………………………………….………..S3-S9**
- **Hydrolytic behavior of Mn-PLY (2) complex…………….....….S10**
  1. **NMR of the ligand of Co-PLY (1), Ni-PLY (3), Al-PLY (4), Fe-PLY (5) complexes:**


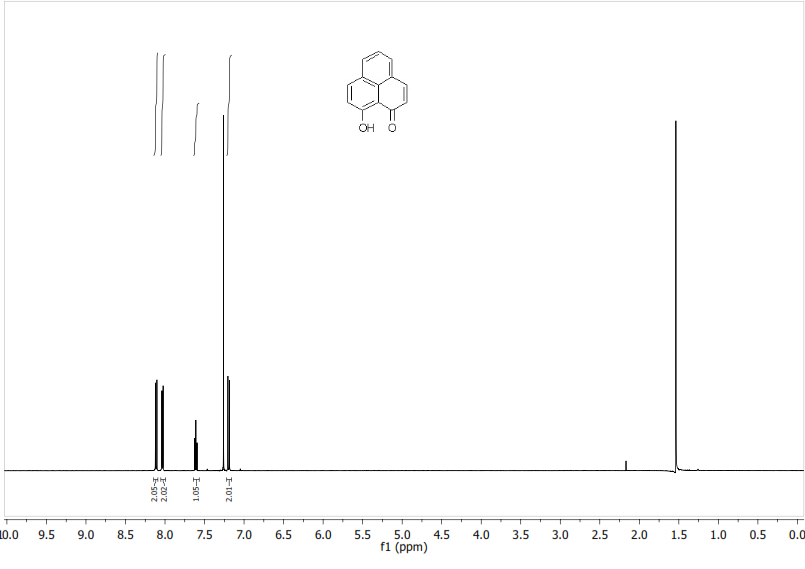


- 1. **NMR of the ligand of Mn-PLY (2) complex:**


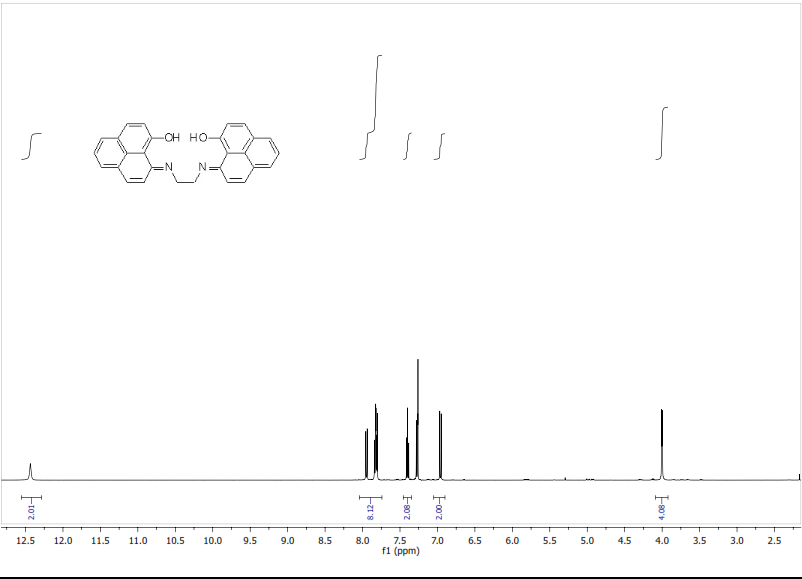


**Synthesis, UV-Vis, MS, and elemental analysis data of the metal PLY (1-5) complexes:**

- 1. **(PLY-O,O)_2_Co(THF)_2_ complex (Co-PLY)(1) :**

Co-PLY complex was prepared by adding a methanolic solution of Co(OAc)_2_.4H_2_O dropwise to hot acetonitrile solution of 9-hydroxyphenalenone followed by refluxing the reaction mixture for 3h to get crystalline precipitate. Recrystallization was performed in a similar way as reported in the literature to get a similar yield(Bhunia et al. 2019).

UV-Vis spectra:

MS:

m/z calc. for C_34_H_30_CoO_6_K [M + K]^+^ 632.10, found 632.20.

Elemental analysis: Analytically Calculated for C_34_H_30_CoO_6_: C: 68.80, H: 5.09; found: C: 68.87, H: 5.13

- 1. **(PLY-O,N)_2_CH_2_CH_2_MnCl (Mn-PLY)(2) :**

The preparation of Mn-PLY **(2)** was carried out by using a pre-synthesized tetra-coordinated Phenalenyl based ligand (H_2_L) with MnCl_2_.4H_2_O in DMF–ethanol mixture (2:1 v/v) at 110^°^C for 12h. The final product was isolated as a black colored micro-crystalline solid with a similar isolated yield(Das et al. 2019).

MS:

m/z calc. for C_28_H_18_MnClN_2_O_2_ [M – Cl]^+^ : m/z 469.07. Found: 469.61

Elemental analysis: Analytically calculated for C_28_H_18_N_2_ClO_2_Mn: C: 66.61; H: 3.59; N: 5.55; found C: 66.70; H: 3.53; N: 5.61,

- 1. **(PLY-O,O)_2_Ni(THF)_2_(Ni-PLY)(3) :**

Ni(OAc)_2_.4H_2_O was reacted with (2 equiv.) of 9-hydroxyphenalenone in methanol at 60^°^C, where by a crystalline precipitate was formed. Then upon recrystallization in dry THF, orange-colored crystals were formed with similar reported yield(Vijaykumar et al. 2018).

MS:

m/z calc. for C_26_H_14_NiO_4_ [M + K]^+^ 486.98, found 486.74.

Elemental analysis: Analytically calculated for C_34_H_30_NiO_6_: C: 68.83; H: 5.09; Found: C: 68.89; H: 5.12.

- 1. **Tris-(9-oxidophenalenone)-aluminum (Al-PLY)(4) :**

In 50 ml of freshly distilled toluene, 9-hydroxyphenalenone (3.25 equiv.) was dissolved, followed by the addition of AlCl_3_ (1 equiv.) and the resultant solution was refluxed overnight. On cooling, a yellow precipitate thus formed was filtered and washed with toluene. The product was further purified by column chromatography and recrystallized from DCM/EtOH. The recrystallized yield was the same as reported(Müller et al. 2013).

MS:

*m/z* calc. for C39H21O6Al [M + Na]^+^ 635.11, found 635.25.

Elemental analysis: Analytically calculated for C39H21O6Al: C: 76.47; and H: 3.45; found C: 76.57; and H: 3.49.

- 1. **Tris-(9-oxidophenalenone)-iron (Fe-PLY)(5) :**

For the preparation of Fe-PLY, (3 equiv.) of 9-hydroxyphenalenone was mixed with 1 equivalent of anhydrous FeCl_3_, refluxed at 60 ^°^C for 3h in MeOH, resulting in a dark precipitate, which was crystallized in DMSO as dark crystals(Pariyar et al. 2015).

MS:

m/z calc. for C_39_H_21_FeO_6_ [M + Na]^+^ 664.06, found 664.14

Elemental analysis: Analytically calculated for C_39_H_21_FeO_6_: C: 73.03, H: 3.30; found: C: 72.98, H: 3.42.

**3.Hydrolytic behavior of Mn-PLY (2) complex:**

We investigated the hydrolytic behavior of the complexes by three methods. Changes in the UV/Vis absorption upon water addition. In a general procedure, the Mn-PLY complex was dissolved in DMSO, and water was added to give a 1:9 water/DMSO solution. The absorbance was monitored for 3 hours (Peri et al. 2009).

**Fig. S1:** UV/Vis absorption over time for Mn-PLY complex upon addition of water to a solution of the complex in DMSO giving a 10% water solution.

Surprisingly, no shift in wavelength was noticed after several hours, ruling out the possibility of hydrolysis for Mn-PLY **2** complex.

**References:**

Bhunia, Mrinal, Sumeet Ranjan Sahoo, Bikash Kumar Shaw, Shefali Vaidya, Anand Pariyar, Gonela Vijaykumar, Debashis Adhikari, and Swadhin K. Mandal. 2019. “Storing Redox Equivalent in the Phenalenyl Backbone towards Catalytic Multi-Electron Reduction.” *Chemical Science* 10 (31): 7433–41. https://doi.org/10.1039/C9SC02057H.

Das, Hari S., Shyamal Das, Kartick Dey, Bhagat Singh, Rahul K. Haridasan, Arpan Das, Jasimuddin Ahmed, and Swadhin K. Mandal. 2019. “Primary Amides to Amines or Nitriles: A Dual Role by a Single Catalyst.” *Chemical Communications* 55 (79): 11868–71. https://doi.org/10.1039/C9CC05856G.

Müller, Sabine, Sabine Steil, Andrea Droghetti, Nicolas Großmann, Velimir Meded, Andrea Magri, Bernhard Schäfer, et al. 2013. “Spin-Dependent Electronic Structure of the Co/Al(OP) _3_ Interface.” *New Journal of Physics* 15 (11): 113054. https://doi.org/10.1088/1367-2630/15/11/113054.

Pariyar, Anand, Gonela Vijaykumar, Mrinal Bhunia, Suman Kr. Dey, Santosh K. Singh, Sreekumar Kurungot, and Swadhin K. Mandal. 2015. “Switching Closed-Shell to Open-Shell Phenalenyl: Toward Designing Electroactive Materials.” *Journal of the American Chemical Society* 137 (18): 5955–60. https://doi.org/10.1021/jacs.5b00272.

Peri, Dani, Sigalit Meker, Michal Shavit, and Edit Y. Tshuva. 2009. “Synthesis, Characterization, Cytotoxicity, and Hydrolytic Behavior of *C* _2_ - and *C* _1_ -Symmetrical Ti ^IV^ Complexes of Tetradentate Diamine Bis(Phenolato) Ligands: A New Class of Antitumor Agents.” *Chemistry - A European Journal* 15 (10): 2403–15. https://doi.org/10.1002/chem.200801310.

Vijaykumar, Gonela, Anand Pariyar, Jasimuddin Ahmed, Bikash Kumar Shaw, Debashis Adhikari, and Swadhin K. Mandal. 2018. “Tuning the Redox Non-Innocence of a Phenalenyl Ligand toward Efficient Nickel-Assisted Catalytic Hydrosilylation.” *Chemical Science* 9 (10): 2817–25. https://doi.org/10.1039/C7SC04687A.
